# Rapid and Robust Generation of Human Cortical Interneurons from Induced Neural Stem Cells

**DOI:** 10.3390/ijms27125194

**Published:** 2026-06-08

**Authors:** Xinwei Zang, Yunqian Guan, Wanting Xing, Zhiguo Chen

**Affiliations:** 1 Cell Therapy Center, Beijing Municipal Geriatric Medical Research Center, Xuanwu Hospital, Capital Medical University, Beijing 100053, China; xinweizang@163.com (X.Z.); guanyunqian@xwhosp.org (Y.G.); xingwanting0821@163.com (W.X.); 2 National Clinical Research Center for Geriatric Diseases, Xuanwu Hospital, Capital Medical University, Beijing 100053, China; 3 Key Laboratory of Neurodegenerative Diseases, Ministry of Education, Xuanwu Hospital, Capital Medical University, Beijing 100053, China; 4 Center of Parkinson’s Disease, Beijing Institute for Brain Disorders, Beijing 100069, China; 5 Center of Neural Injury and Repair, Beijing Institute for Brain Disorders, Beijing 100069, China

**Keywords:** human-induced neural stem cells, cortical interneurons, GABA, rapid differentiation, interneuron transplantation, cell survival, synaptic integration, neural repair

## Abstract

Current protocols for generating cortical interneurons from human pluripotent stem cells are hindered by slow differentiation kinetics and poor reproducibility across cell lines. Here, we present a defined small-molecule-based strategy that efficiently directs human-induced neural stem cells (hiNSCs) toward cortical GABAergic interneurons within 14–18 days, which is substantially faster than conventional methods. Short-term dual-SMAD and WNT inhibition rapidly commits hiNSCs to an interneuron progenitor fate, reaching transcriptional states equivalent to those obtained with prolonged protocols. Prolonged activation of Sonic Hedgehog (via SAG) further enhances lineage specification, markedly upregulating NKX2.1, FOXG1, GABA, somatostatin (SST), and parvalbumin (PV) expression, and enriching pathways associated with early functional maturation. Importantly, RNA-sequencing reveals that under identical induction conditions, hiNSCs differentiate more rapidly and homogeneously than human-induced pluripotent stem cells (hiPSCs), which exhibit broader, less lineage-focused transcriptional trajectories. This differentiation strategy is highly reproducible across four genetically distinct hiNSC lines, with minimal off-target populations. Functionally, hiNSC-derived cortical interneurons display robust migratory behavior, produce abundant GABA, and survive transplantation into the adult mouse hippocampus, where they extend processes and form synapse-like structures. These findings establish a rapid, scalable, and robust approach for generating human cortical interneurons, supporting their safety and integration potential as a foundation for future cell replacement strategies in neurological disorders.

## 1. Introduction

Human pluripotent stem cells (hiPSCs) represent powerful tools for investigating human development and disease, as well as for applications in regenerative medicine. Consequently, hiPSCs and human embryonic stem cells (hESCs) are widely utilized as the initiating cell sources for differentiation protocols. Given the paucity of effective therapies for numerous neurodegenerative and neuropsychiatric disorders, coupled with the availability of protocols that effectively direct in vitro neuronal specification, the derivation of central nervous system (CNS) lineages from hiPSCs has garnered particular attention [1,2]. Early investigations utilizing hiPSCs primarily focused on neurodegenerative diseases characterized by the selective vulnerability of specific neuronal subtypes, such as Parkinson’s disease [3,4], amyotrophic lateral sclerosis [5,6], spinal muscular atrophy (SMA) [6,7], and epilepsy [8,9]. More recently, complex psychiatric disorders, including depressive disorders [10], schizophrenia [11,12,13], and autism-related syndromes [14,15,16], have also been explored using hiPSC- and hESC-derived CNS lineages.

Despite considerable progress in establishing protocols for deriving cortical neurons [17,18,19], modeling human cortical interneuron development in vitro is of particular interest due to significant interspecies differences among mammals [20,21]. Furthermore, the vast diversity of cortical interneuron subtypes presents additional challenges for modeling their differentiation using human cells in vitro [22]. However, the efficiency of generating cortical interneurons remains low, typically requiring over 35 days [23,24,25,26,27,28]. Moreover, the elimination of contaminating cells and proliferating progenitors remains problematic, posing a potential risk of tumorigenicity in the final product [1,29].

Human-induced neural stem cells (hiNSCs), generated via the direct reprogramming of peripheral blood mononuclear cells (PBMCs), represent homogeneous, lineage-primed cell lines that bypass early pluripotent patterning stages. This developmental positioning makes them an ideal starting population for rapid and directed generation of human interneuron progenitors. Notably, dopaminergic neural progenitors differentiated from these hiNSCs have already been successfully applied in the clinical treatment of patients with Parkinson’s disease, with preliminary results supporting their safety and therapeutic potential [30,31].

During CNS development, the precise patterning of neural progenitors is governed by the coordinated activity of several key signaling pathways. The transforming growth factor-β (TGF-β) and bone morphogenetic protein (BMP) pathways promote non-neural ectodermal and mesodermal fates; thus, their inhibition (dual SMAD inhibition) is a canonical approach to enforce neural induction [2,32]. The Wingless/Integration (WNT) pathway exhibits a posterior-to-anterior gradient in the developing neural tube, where high WNT activity specifies posterior fates while its inhibition is required for forebrain identity acquisition [32,33]. Sonic Hedgehog (SHH) signaling, in contrast, establishes ventral patterning along the dorsoventral axis; its graded activity is essential for specifying distinct interneuron subtypes from the medial ganglionic eminence [34,35,36]. The MEK/ERK pathway, downstream of fibroblast growth factor (FGF) signaling, supports neural progenitor proliferation and survival while suppressing non-neural differentiation [9,37,38]. Lastly, the Notch pathway maintains the neural progenitor pool via lateral inhibition, and its blockade promotes neuronal differentiation [39].

While multiple studies have demonstrated successful differentiation of cortical interneurons from hiPSCs and hESCs via identical small-molecule cocktails, the feasibility of adapting these strategies to hiNSCs remains poorly defined. Building upon these developmental principles, we modulated a small-molecule-based strategy to induce hiNSCs into cortical interneurons. In addition to the modulation of SMAD, WNT, SHH, MEK, and Notch signaling, we explored culture configurations, including three-dimensional differentiation, which more closely mimics in vivo tissue architecture and facilitates extracellular matrix formation [40]. Such conditions may stabilize progenitor proliferation, support synchronous differentiation, and provide instructive microenvironmental cues for subtype specification [41]. By integrating pathway modulation with culture system optimization, our approach establishes a framework for the rapid, reproducible, and scalable generation of cortical interneuron progenitors from hiNSCs.

## 2. Results

### 2.1. Short-Term SMAD and WNT Inhibition Efficiently Drives hiNSC Commitment to an Interneuron Lineage

**Figure 1:**
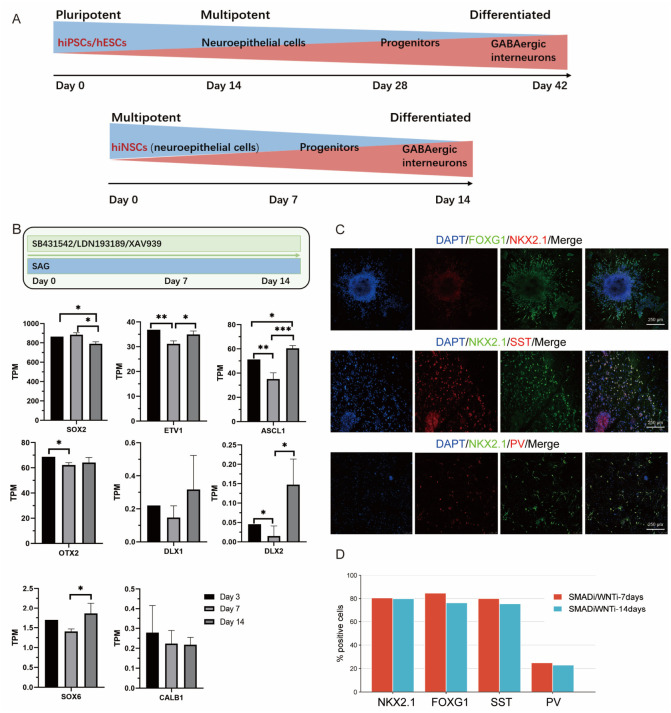
Short-term SMAD and WNT inhibition efficiently induces interneuron progenitor specification. (**A**) Workflow of the differentiation strategy of human-induced pluripotent stem cells/ human embryonic stem cells (hiPSCs/hESCs) and human-induced neural stem cells (hiNSCs). (**B**) Schematic overview of dual SMAD and WNT inhibitor timing, and RNA-seq analysis of hiNSCs undergoing short-term (3-day and 7-day) or extended (14-day) SMAD and WNT inhibition. (**C**,**D**) Representative immunofluorescence images of cells (**C**) and quantification analysis of interneuron marker-positive cells (**D**) on day 14 of differentiation. Cells were treated with dual SMAD and WNT inhibition for 14 days and stained for interneuron progenitor markers. Scale bars = 250 μm. Data are presented as mean ± SEM from three independent differentiation experiments. * *p* < 0.05, ** *p* < 0.01, *** *p* < 0.001. SMADi/WNTi: SMAD and WNT inhibition.

The first step in recapitulating cortical interneuron development is the robust induction of forebrain progenitors. Dual SMAD inhibition—blocking both TGFβ and BMP signaling pathways—effectively converts hiPSCs into neuroepithelial cells [32,33,42]. Comparison of differentiation workflows revealed that hiNSCs follow a markedly accelerated induction timeline relative to hiPSCs/hESCs (Figure 1A). To determine whether the temporal window of dual SMAD inhibition (using LDN193189 and SB431542) and WNT inhibition (XAV939) influences the commitment of hiNSCs toward an inhibitory interneuron fate, we performed transcriptomic profiling of hiNSCs exposed to different durations of inhibitor treatment (Figure 1B).

**Figure 2:**
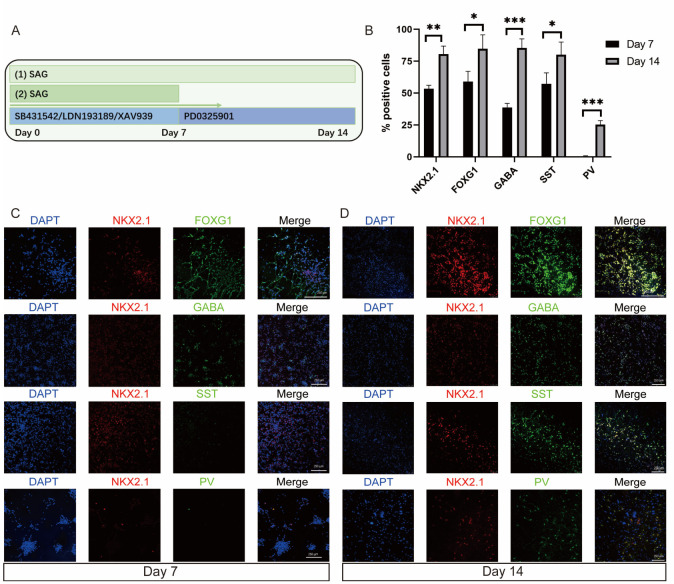
Sonic Hedgehog (SHH)-dependent specification of cortical GABAergic progenitors from hiNSCs. (**A**) Schematic of the differentiation protocol for hiNSCs subjected to short-term (7-day) or extended (14-day) SHH activation. (**B**) Quantification of the percentage of NKX2.1^+^, FOXG1^+^, GABA^+^, somatostatin^+^ (SST^+^), and parvalbumin^+^ (PV^+^) cells at day 20 of differentiation. Data are shown as mean ± s.e.m., n = 3 independent differentiation experiments. (**C**,**D**) Representative immunofluorescence images of cortical interneurons immunostained for NKX2.1, FOXG1, GABA, SST and PV with 7-day SAG (**C**) and 14-day SAG (**D**) treatment. Scale bars = 250 μm. * *p* < 0.05, ** *p* < 0.01, *** *p* < 0.001.

RNA-seq analysis revealed a prominent transcriptional inflection point at differentiation day 7 under SMAD and WNT inhibition conditions. At this timepoint, the neural progenitor marker *SOX2* reached peak expression and subsequently declined, while early migratory genes *DLX1/2* and *CXCR4* exhibited simultaneous downward inflection points. Concurrently, genes associated with interneuron progenitors and ventral forebrain identity, including *ASCL1*, *OTX2*, *SOX6*, and *ETV1*, displayed coordinated changes (Figure 1B), indicating dynamic transcriptional progression from neural progenitors toward inhibitory interneuron lineage commitment. To further validate the biological outcome of the optimized protocol, we performed immunostaining analysis at day 14 of differentiation. Short-term (7-day) dual SMAD and WNT inhibition generated proportions of inhibitory interneuron progenitors comparable to those obtained using sustained 14-day inhibition, and quantitative analysis revealed no statistically significant difference between the two conditions (Figure 1C,D and Figure 2D). Collectively, the RNA-seq results suggested day 7 as a potential transition point suitable for optimizing the duration of dual SMAD and WNT inhibition in hiNSCs.

Regarding the withdrawal of dual SMAD and WNT inhibitors after day 7 and continued differentiation culture, transcriptomic analysis revealed progressive enrichment of cortical interneuron-associated gene signatures (Supplemental Figure S1A,B). Among the dynamically regulated genes were *MDK*, *CRABP1*, *GPNH*, and *MAF*, together with the tangential migration-associated gene *ERBB4*, as well as genes related to inhibitory neurotransmitter synthesis and somatostatin^+^ (SST^+^)/parvalbumin^+^ (PV^+^) progenitor identity, including *GAD1*, *MAFB*, and *SATB1* (Supplemental Figure S1B). In contrast, the off-target neuronal marker *CALB1* showed a declining trend during this stage (Supplemental Figure S1B). These transcriptional dynamics suggested progressive lineage specification toward inhibitory interneurons. Heatmap analysis across different differentiation timepoints further confirmed the stepwise acquisition of cortical interneuron-associated molecular features by hiNSCs (Supplemental Figure S1C). To further assess the robustness and reproducibility of the optimized differentiation strategy, four independent donor-derived hiNSC lines were subjected to the 7-day protocol. Expression analysis demonstrated highly consistent levels of representative stage-specific genes across all four donor-derived hiNSC lines at day 14 of differentiation (Supplemental Figure S1D), supporting the inter-line reproducibility of the protocol. Overall, these findings indicate that short-term dual SMAD and WNT inhibition is sufficient to support efficient interneuron lineage induction from hiNSCs while reducing the duration of early patterning conditions relative to conventional prolonged inhibition strategies.

### 2.2. Prolonged SHH Activation Enhances Cortical Interneuron Fate Commitment and Functional Maturation

Ventral forebrain identity is induced by SHH signaling. Next, to determine the impact of the SHH activation window on cortical interneuron generation, we compared 7-day and 14-day SHH activation windows under a constant SMAD/WNT/MEK inhibition background (Figure 2A). Prolonged (14-day) SHH pathway activation markedly enhanced both the purity and batch-to-batch reproducibility of cortical inhibitory interneuron progenitors compared to short-term SHH activation (Figure 2B–D). At the end of differentiation, extended SHH activation yielded a robust ventral forebrain identity, with NKX2.1^+^ cells reaching 80.6 ± 3.7% versus 53.4 ± 1.6% in the short-term group, and FOXG1^+^ cells reaching 84.7 ± 6.4% versus 59.2 ± 4.6%. Markers of inhibitory neuronal differentiation were similarly elevated, including GABA^+^ cells (85.3 ± 4.2% versus 38.6 ± 1.8%), SST^+^ cells (80.0 ± 5.8% versus 57.3 ± 5.0%), and PV^+^ cells (25.3 ± 1.8% versus 0.5 ± 0.2%). These improvements were consistently observed across three independent differentiation batches (Figure 2B).

To reveal functional changes in gene expression patterns during neural stem cell differentiation, we performed Gene Set Enrichment Analysis (GSEA) based on RNA-seq data (Supplemental Figure S2). Under the Cellular Component (CC) category, significantly enriched pathways primarily involved neuronal structure and morphogenesis, including “axon part,” “dendrite,” and “neuron projection development.” This indicates that neural stem cells initiate neuronal process formation and cytoskeletal remodeling during early differentiation, establishing the foundation for neuronal morphological differentiation (Supplemental Figure S2A). In KEGG pathway analysis, enriched signaling pathways mainly included neural signaling-related pathways such as “calcium signaling pathway,” “neuroactive ligand–receptor interaction,” “morphine/nicotine addiction,” as well as “ribosome biogenesis in eukaryotes.” Upregulation of these pathways reflects that late-stage differentiated cells progressively acquire neuronal characteristics, manifested by neurotransmitter receptor activation, calcium signaling-mediated enhanced excitability, and active protein synthesis (Supplemental Figure S2B).

Collectively, these results demonstrate that 14-day SHH activation not only enhances cortical interneuron identity commitment but also promotes cellular transition from structural maturity to a functional neuronal state during later stages.

### 2.3. Impact of Adherent and Shaker Culture on Cortical GABAergic Progenitor Expansion and Differentiation

**Figure 3:**
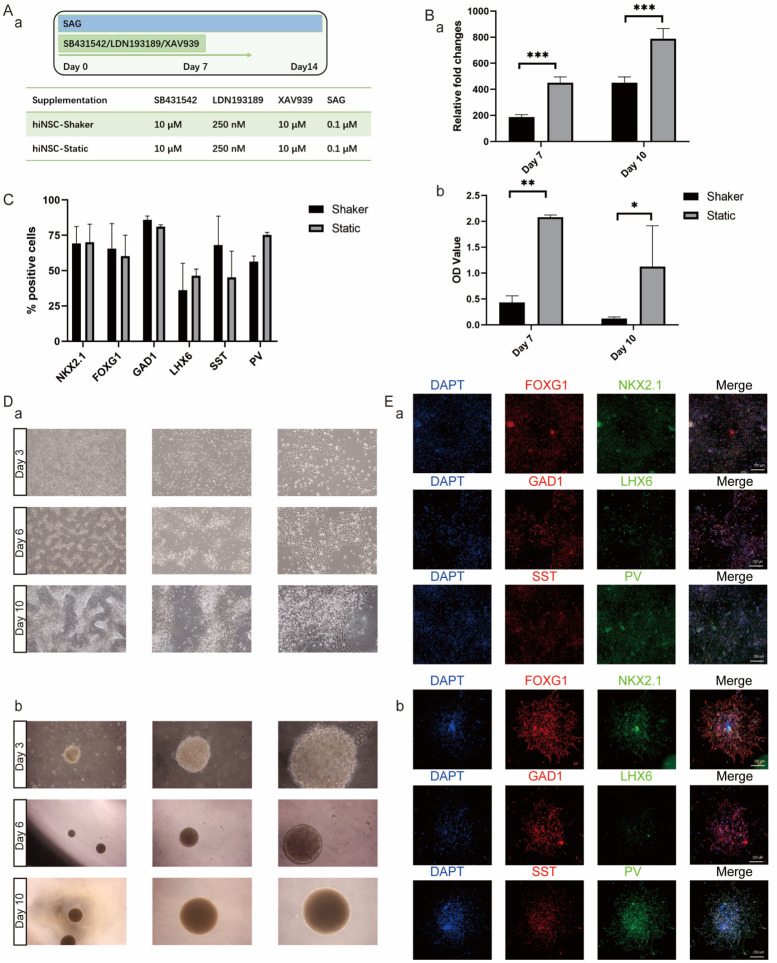
Effects of culture format on hiNSC differentiation into cortical progenitors and cortical interneurons. (**A**) Schematic overview of the differentiation protocol for hiNSCs under adherent and orbital shaker culture conditions. (**B**) Cell proliferation measured by direct cell counts (**a**) and CCK-8 assays (**b**) across different culture conditions. Data are presented as mean ± s.e.m., n = 3 independent differentiation experiments. (**C**) Quantification of the proportion of NKX2.1^+^, FOXG1^+^, GAD1^+^, LHX6^+^, SST^+^, and PV^+^ cells at day 10 of differentiation. Data are shown as mean ± s.e.m., n = 3 independent differentiation experiments. (**D**) Brightfield images of cells cultured under static (**a**) and shaker (**b**) conditions. Images were acquired at 40×, 100×, and 200× magnification from left to right. (**E**) Representative immunofluorescence images of cells in static (**a**) and shaker (**b**) culture conditions. Scale bars = 250 μm. * *p* < 0.05, ** *p* < 0.01, *** *p* < 0.001.

For efficient use of hiNSC-derived cortical interneurons, it is imperative to derive them at a scale sufficient for industrial applications in drug screening or cell therapy. Thus, we tested the effect of different culture methods on the expansion kinetics and differentiation progression of cortical interneuron progenitors. Cortical GABAergic progenitor cells were induced for 14 days according to our previously mentioned protocol (Figure 3A).

RNA-seq analysis further revealed differences in differentiation kinetics between the two conditions (Supplemental Figure S3A). At day 10 of induction, cells cultured under adherent conditions displayed gene expression profiles corresponding to a more advanced differentiation stage compared to cells in orbital shaker culture (Supplemental Figure S3B,C). The Biological Process category was similarly enriched in pathways related to “nervous system development,” “generation of neurons,” “neuron differentiation,” “neurogenesis,” and “neuron development” (Supplemental Figure S3B). GO enrichment analysis of upregulated genes in adherent cultures revealed significant enrichment in Cellular Component categories associated with neuronal structure and morphogenesis, including anchoring junction, somatodendritic compartment, cell junction, neuronal cell body, synapse, and neuron projection (Supplemental Figure S3C). Despite differences in early differentiation kinetics, both culture formats ultimately generated highly homogeneous cortical GABAergic progenitor populations.

CCK-8 assays and direct cell counting indicated that cells cultured under static conditions exhibited an earlier onset of rapid expansion compared with those maintained in orbital shaker culture. (Figure 3B). Immunofluorescence analysis revealed uniform expression of canonical cortical GABAergic progenitor markers in both groups (Figure 3C–E). Cells from spinner culture generated homogeneous populations of cortical interneurons, shown by robust expression of NKX2.1 (74.8 ± 7.0% versus 70.7 ± 12.8% of total cells), FOXG1 (55.3 ± 0.7% versus 67.8 ± 6.9%), and LHX6—directly activated by NKX2.1—reaching 36.1 ± 13.5% versus 46.5 ± 3.3%. GAD1-positive cells exceeded 85.8 ± 2.0% versus 81.1 ± 1.0%, among which some cells expressed more mature interneuron markers SST^+^ (68.0 ± 14.5% versus 45.3 ± 13.1% of total cells) and PV^+^ (56.4 ± 2.7% versus 75.3 ± 1.3% of total cells) (Figure 3C). Notably, the proportion of SST-positive cells was slightly higher in suspension culture, while static cultures showed a higher percentage of PV-subtype inhibitory interneurons (Figure 3C), although neither difference reached statistical significance. In summary, different culture formats effectively supported the generation of cortical progenitors and subsequent cortical interneurons.

### 2.4. Robustness of the Differentiation Protocol Across Multiple hiNSC Lines

**Figure 4:**
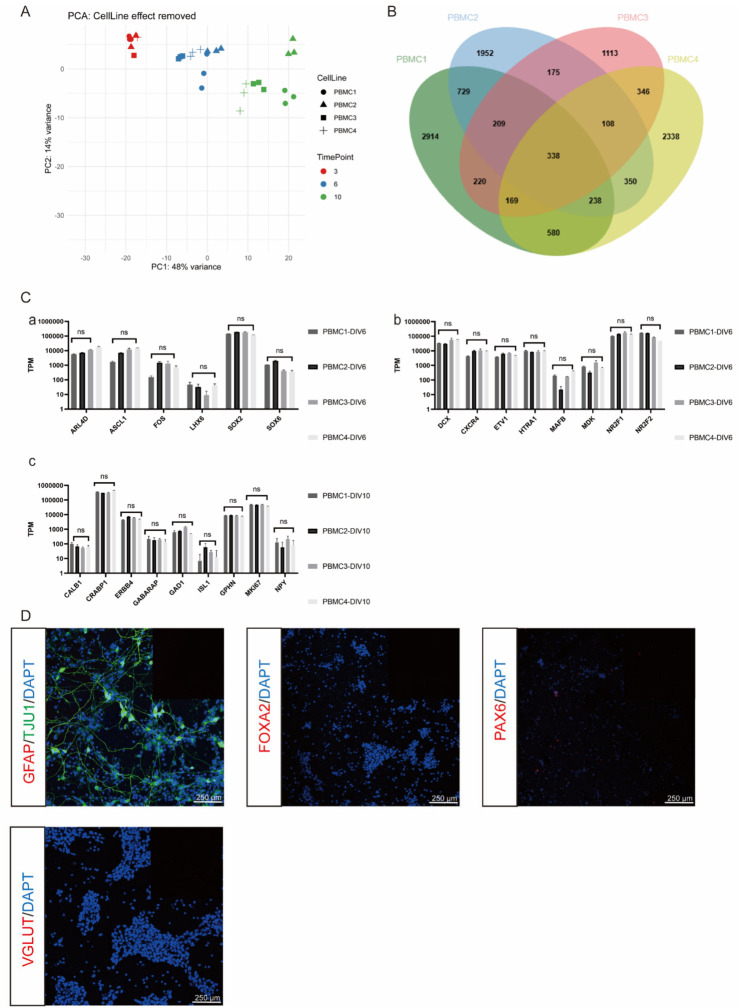
Differentiation reproducibility across independent hiNSC lines. PBMC1–4 denotes hiNSC lines derived from four independent PBMC donors. (**A**) Principal component analysis (PCA) of RNA-seq data from four hiNSC lines at days 3, 6, and 10 of differentiation. Samples cluster primarily by differentiation stage. (**B**) Venn diagram showing shared and unique expressed genes among the four hiNSC lines at day 10. (**C**) Heatmap of core lineage genes at day 10 across the four hiNSC lines, including neural progenitor-associated genes (**a**,**b**) and interneuron lineage-related genes (**c**). Colors represent normalized expression levels. (**D**) Representative immunofluorescence images showing the absence of major off-target lineage markers. Scale bar = 250 μm. PBMC: Peripheral blood mononuclear cells.

To evaluate whether this differentiation protocol induces similar transcriptional dynamics across different genetic backgrounds, we applied the same differentiation procedure to three additional independent hiNSC lines. Four hiNSC lines were induced to differentiate in static culture according to the protocol described above. Principal component analysis (PCA) showed that samples from different cell lines but at the same differentiation timepoint (days 3, 6 and 10) exhibited tight clustering, whereas samples from different timepoints were clearly separated along the principal component axes (Figure 4A). Venn diagram analysis revealed that 45.5–52.4% of differentially expressed genes were shared among all four cell lines (Figure 4B), suggesting that despite cell line-specific background differences, they share stable core transcriptional features. Further visualization of key neural differentiation-associated genes demonstrated highly consistent temporal expression trends across all four lines (Figure 4C), unaffected by cell line-specific variation.

These results indicate that transcriptional differences are primarily driven by differentiation stage rather than cell line origin, demonstrating good reproducibility and comparability of the differentiation process across diverse genetic backgrounds. Additionally, very few other neural cell types were generated, such as astrocytes (GFAP^+^), midbrain marker FOXA2^+^ cells, dorsal telencephalic neural progenitor marker PAX6^+^ cells, or excitatory glutamatergic neurons (VGLUT^+^) (Figure 4D).

### 2.5. Differential Characteristics Between hiNSCs and hiPSCs Under Identical Differentiation Conditions

**Figure 5:**
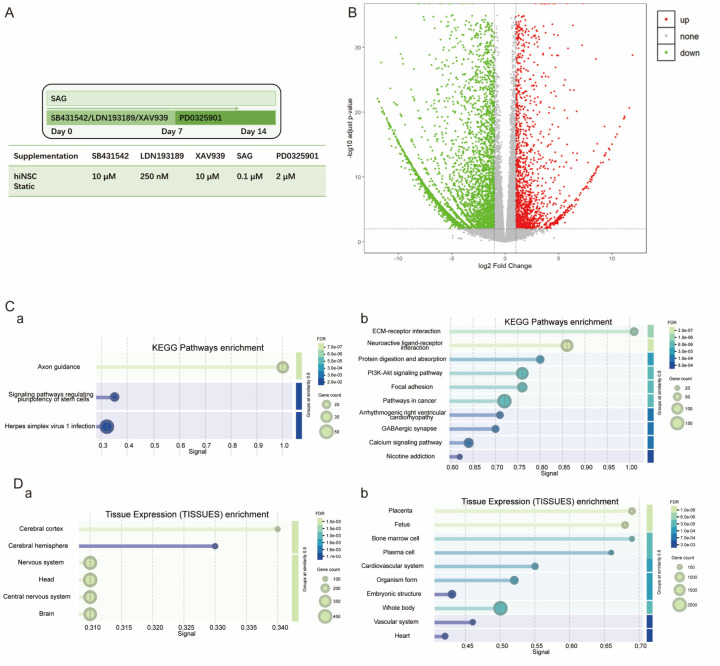
Comparative transcriptional and lineage characteristics of hiNSCs and hiPSCs under identical induction conditions. (**A**,**B**) Schematic overview of the induction protocol (**A**) and volcano plot (**B**) showing differentially expressed genes (DEGs) between hiNSC-derived and hiPSC-derived cells at day 14 of induction. Red: upregulated genes in hiNSCs (Log2 FC > 1, *p* < 0.05); green: downregulated genes in hiNSCs. (**C**,**D**) KEGG pathway enrichment (**C**) and tissue-specific expression analysis (**D**) for hiNSC-derived cells (**a**) and hiPSC-derived cells (**b**) at day 14 of induction.

Building on these findings, we further compared the transcriptional responses of hiNSCs versus hiPSCs under the same differentiation protocol (Figure 5A). Transcriptomic profiling at day 14 showed that genes upregulated in hiNSC-derived cultures, relative to hiPSC-derived cultures, were significantly enriched in GO and KEGG terms associated with neuronal projection development, axon guidance, and central nervous system-related processes. (Supplemental Figure S4A,B). Tissue-specific expression analysis further showed that gene expression patterns highly corresponded to cerebral cortex and central nervous system regions, suggesting focused and defined differentiation trajectories (Figure 5(Da)). In contrast, at the same timepoint, hiPSCs displayed a more dispersed pattern of differentially expressed genes (DEGs) involving multiple cell types and tissue-associated pathways (Figure 5(Cb,Db)), suggesting that their differentiation state may contain more non-specific or heterogeneous cellular components.

Overall, hiNSCs formed a more directed and highly neural lineage-committed transcriptional state by day 10 of induction, whereas hiPSCs exhibited more heterogeneous differentiation characteristics at this stage. Therefore, hiNSCs represent a more homogeneous starting cell source better suited for rapid and efficient generation of inhibitory interneurons.

### 2.6. hiNSC-Derived GABAergic Interneurons Exhibit Migratory Properties and Produce Abundant GABA Neurotransmitter

**Figure 6:**
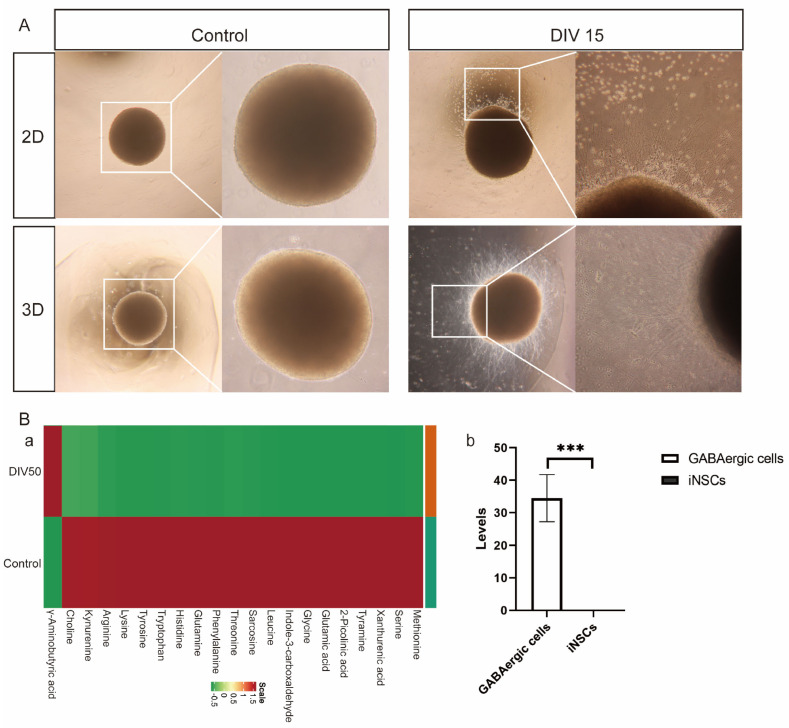
Characterization of hiNSC-derived cortical interneurons in vitro. (**A**) Migration behavior of hiNSC-derived cortical interneurons assessed in two-dimensional and three-dimensional culture formats. Representative images show cells extending processes and migrating outward from aggregates or cluster edges. Images were acquired at 40× and 100× magnification from left to right. (**B**) GABA expression in hiNSC-derived interneurons at day 60 of differentiation. Heatmap (**a**) and bar chart (**b**) illustrating GABA-related marker expression levels. *** *p* < 0.001. DIV: days in vitro.

During embryonic development, interneurons exhibit robust tangential migration toward the developing cortex. hiNSC-derived GABAergic interneurons recapitulate such migratory characteristics (Figure 6A). Following extended plating and coating time, cells migrated radially outward from cell clusters. To confirm the functionality of hiNSC-derived GABAergic cells, we performed multiple reaction monitoring (MRM) analysis of culture supernatants following high-potassium stimulation and detected abundant release of γ-aminobutyric acid (GABA) (Figure 6(Bb)), with no detectable serotonin, succinic acid, or acetylcholine (Figure 6(Ba)).

### 2.7. hiNSC-Derived GABAergic Interneurons Migrate Efficiently and Functionally Integrate into the Adult Mouse Hippocampus

**Figure 7:**
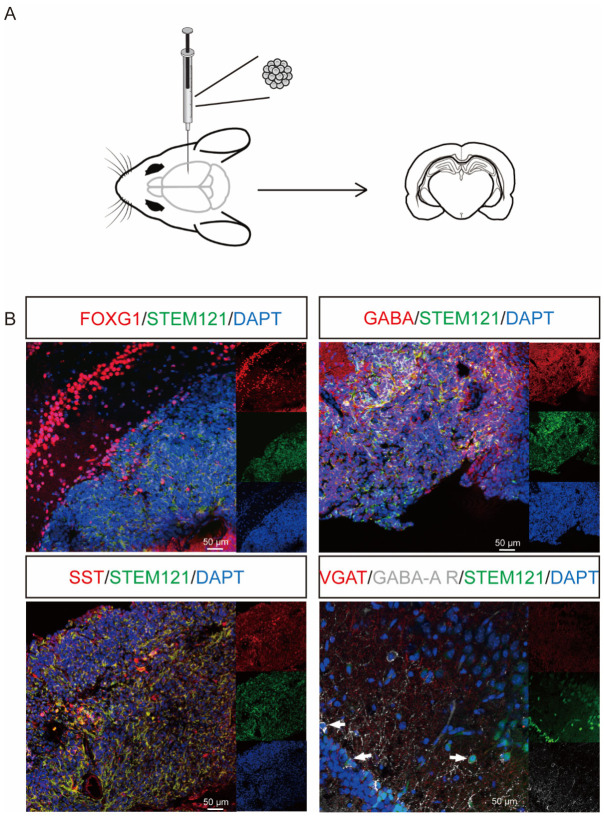
Characterization of hiNSC-derived cortical interneurons in vivo. (**A**) Schematic representation of experimental design. (**B**) Survival and morphological features of hiNSC-derived interneurons transplanted into the adult mouse hippocampus 30 days post-transplantation. Representative images show human donor cells expressing cortical interneuron markers (FOXG1, GABA, SST), along with extended neuronal processes and presynaptic puncta (white arrows) apposed to host neurons. Scale bars = 50 μm.

Cell transplantation, particularly cortical interneuron transplantation, has been shown to ameliorate symptoms in various preclinical models of central nervous system disorders, including epilepsy, Parkinson’s disease, neuropathic pain, schizophrenia, and cognitive deficits. To determine whether transplanted inhibitory interneuron progenitors could survive and continue to differentiate in vivo, we transplanted day 15-differentiated cell aggregates (1 × 10^6^ cells per mouse) into the hippocampus of NOD/SCID mice. After 4 weeks, mice were sacrificed for analysis, and robust surviving cells were detected in the brains of mice that received day 15 hiNSC-derived GABAergic progenitor transplants (Figure 7A). Specific GABAergic interneuron subtype markers, including FOXG1, GABA, and SST, were readily detectable in the grafted cells. Furthermore, triple immunofluorescence analysis localizing human-specific VGAT (a presynaptic protein in graft-derived neurons), the postsynaptic receptor protein GABA-A receptor (GABA-AR), and the human nuclear marker Stem121, demonstrated that graft-derived neurons formed putative synaptic contacts with host cells in the stratum radiatum (Figure 7B). Thus, transplanted GABAergic interneurons appeared to achieve synaptic integration with host neurons. This synaptic connectivity underscores the substantial translational potential of hiNSC-derived inhibitory interneurons.

## 3. Discussion

Many therapies that demonstrate efficacy in animal models fail to replicate their effects in human clinical trials [43,44], highlighting the urgent need to develop novel therapeutics using authentic human tissue. Large-scale, reliable, and efficient generation of human cortical inhibitory interneurons is of paramount importance for elucidating their roles in development and disease, constructing physiologically relevant disease models, and developing cell replacement-based therapeutic strategies.

However, existing protocols for generating cortical interneurons from hiPSCs or hESCs typically require induction periods exceeding 30 days, with maturation of specific subtypes demanding even longer durations (60 days or more) [23,24,26,27]. More critically, hiPSCs and hESCs possess robust potential to differentiate into all three germ layers, presenting persistent safety concerns related to residual pluripotent cells [25,29,42]. Developing an induction strategy with shortened timelines, enhanced purity, reduced safety risks, and stable output for large-scale production holds critical value for accelerating neurodevelopmental disease research and cell therapy translation [1,45]. Disorders such as schizophrenia [11,12,13], pathological epileptic seizure activity [8,9], autism spectrum disorder [14,15,16], Timothy syndrome [46], and depressive disorders [10] urgently require such advanced platforms. Previous studies have provided numerous methods for generating cortical interneurons from hiPSCs and hESCs with varying efficiencies, some employing genetic modifications [23,24,47], while others utilize developmentally relevant signaling molecules [26,27,33,48]. However, current protocols for generating cortical interneurons remain limited in their efficiency, simplicity, and reproducibility, and few are suitable for transplantation-oriented applications.

Forebrain and anterior fate specification is considered the default program during neural differentiation of hiPSCs [2,17,49,50]. Nevertheless, not all cell lines adopt proneural fate with equivalent efficiency [19,51,52]. In neural induction, inhibition of BMP/TGF-β and WNT signaling constitutes the canonical approach for acquiring forebrain identity [9,32]. In most protocols for directing hiPSCs or hESCs toward inhibitory interneurons, SMAD and WNT inhibitors are typically applied for approximately 14 days [9,27]. Considering that hiNSCs already reside at a downstream stage of hiPSC and hESC differentiation, we hypothesized that the temporal window of SMAD and WNT inhibitor exposure could be further shortened without compromising differentiation efficiency.

While similar small-molecule cocktails have previously been applied to differentiate hiPSCs/ESCs into cortical interneurons [24,25,33], the applicability of these paradigms to hiNSCs—which already possess neural lineage commitment and developmental priming—has not been systematically investigated. Therefore, we systematically examined the effects of dual SMAD and WNT inhibition and SHH pathway activation on directed differentiation. Compared with hiPSCs, hiNSCs appeared more responsive to patterning signals under the same induction conditions. Specifically, brief dual SMAD and WNT inhibition (7 days) was sufficient to robustly induce hiNSCs to acquire stable ventral forebrain identity, whereas many hiPSC/hESC-based interneuron differentiation protocols require 14 days or more than 30 days of neural induction before subsequent ventral specification [9,42]. This suggests that hiNSCs occupy a downstream position in the neural lineage relative to hiPSCs, harboring developmental memory and lineage priming that may bias neural fate commitment toward ventral forebrain lineages, thereby significantly abbreviating the induction period. For example, previous studies using hiPSCs/hESCs reported that generation of SST^+^ and PV^+^ cortical interneuron subtypes generally required prolonged differentiation and maturation periods extending beyond 30 days [9,27,28,33].

It is well established that PV^+^ and SST^+^ cortical interneurons are specified by different levels of SHH and are produced in different medial ganglionic eminence regions [27,33]. Reports on interneuron generation from hiPSCs typically employ ventral SHH activation [53,54], though the optimal timing of activation varies across studies. We therefore compared exposure durations to the SHH pathway agonist SAG. Sustained 14-day SAG treatment generates a higher proportion of target cells, whereas 7-day treatment proves relatively insufficient. This outcome is consistent with the classical SHH-dependent ventralization process. RNA-seq results corroborate the facilitative effect of extended SAG treatment on differentiation. Collectively, these findings indicate that prolonged SHH stimulation reinforces ventral fate commitment and is associated with transcriptional programs related to neuronal maturation, suggesting tighter coupling between regionalization and maturation in hiNSCs. Following 14-day SHH activation, the majority of cells co-expressed the ventral forebrain markers NKX2.1 and FOXG1 at the end of differentiation. Over 80% of cells were GABA^+^ and SST^+^. In contrast to hiPSC/hESC-based protocols such as those of Maroof et al. [33] and Nicholas et al. [27] (approximately 70% GABA^+^ and 40% SST^+^), which inevitably generate off-target cells due to pluripotency [33]. As a result, our optimized protocol consistently generated cortical interneurons from hiNSCs within 14 days, achieving comparable or higher enrichment of inhibitory interneuron populations while minimizing off-target differentiation.

Building upon these observations, we further postulated whether the heightened sensitivity of hiNSCs to regionalization signals extends to their responsiveness to external culture environments. To address this, we tested different culture systems for inducing cortical interneurons and determined the impact of traditional static adherent culture versus suspension culture on shakers, inducing differentiated cell fate. hiNSCs cultured under static adherent conditions exhibited greater propensity toward PV subtype interneuron differentiation. Additionally, possibly due to superior access to nutrients and oxygen in monolayer culture, cells in static culture demonstrated accelerated proliferation and differentiation kinetics compared with shaker culture environments. This finding contrasts with a previous report by Li et al. [54]. Conversely, suspension-cultured organoids permit interactions among multiple cell types, generating relatively higher proportions of SST subtype cells without compromising overall purity. These differences suggest that the ventral forebrain-directed differentiation process of hiNSCs possesses plasticity toward microenvironmental cues, whereby distinct culture systems may be leveraged to modulate the generation of specific inhibitory interneuron subpopulations, providing novel strategies for future subtype-specific replacement therapies.

Cross-individual reproducibility constitutes a critical metric upon which any cell therapy or drug screening platform depends [9,29]. In this study, we systematically evaluated the reproducibility of the optimized differentiation protocol across hiNSCs lines derived from different individuals. Results demonstrate that this protocol stably induces high proportions of cortical inhibitory interneuron progenitors with robust consistency and reproducibility. Even when donor-derived hiNSCs exhibit variability in basal transcriptional profiles, their differentiation trajectories during induction remain highly concordant. PCA reveals that expression patterns are primarily driven by “differentiation stage” rather than “genetic background,” potentially attributable to the protocol’s concurrent inhibition of SMAD, WNT, and MEK pathways during early stages, thereby rapidly channeling hiNSCs from diverse genetic origins into similar neurodevelopmental trajectories, enabling more uniform ventral forebrain responses during subsequent SHH activation. These results indicate that this protocol not only operates stably within individual hiNSC lines but also maintains high efficiency and predictability across multiple cell lines. This will provide more reliable cell sources for downstream disease modeling, drug screening, and even cell therapy applications, while significantly reducing technical noise arising from experimental batch variations.

Although numerous studies have reported efficient cortical interneuron differentiation from hiPSCs and hESCs using similar small-molecule combinations [9,55], hiNSCs represent a developmentally distinct starting population compared with pluripotent stem cells. hiNSCs are directly reprogrammed from peripheral blood mononuclear cells without transitioning through the hiPSC stage, representing homogeneous, highly stable monoclonal stem cell lines [30,56]. hiNSCs share several transcriptional and developmental features with early neuroepithelial cells, possessing the potential to differentiate into specific cell types across any brain region, thereby holding significant clinical application promise. Direct comparison of hiNSCs and hiPSCs under identical differentiation conditions revealed inherent differences in developmental stage, fate preference, and differentiation kinetics between the two cell types. Under equivalent SMAD/WNT inhibition and SHH activation conditions, hiNSCs more readily acquire ventral forebrain specification, whereas hiPSCs are prone to off-target differentiation. This may reflect the heightened sensitivity of pluripotent state cells to pathway perturbations, whereas the developmental memory of hiNSCs confers stronger fate constraints within the nervous system. Notably, hiNSCs enter functional maturation-associated transcriptional programs earlier than hiPSCs, suggesting a comparatively accelerated differentiation trajectory. hiNSCs directly enter the ventral forebrain program from a slightly matured, lineage-primed neural state, resulting in more rapid differentiation responses and more focused trajectories. In summary, hiNSCs exhibit advantages in temporal efficiency, fate stability, and functional maturation, and thus represent a suitable cell source for experimental systems requiring rapid and low-variance differentiation.

It is well established that human cortical interneurons derived from hiPSCs and hESCs follow protracted in vivo developmental timelines [24,26,27]. Our induction protocol generates cortical interneurons that undergo sustained maturation, similar to normal developmental processes [24,26,27]. Importantly, transplantation of hiNSC-derived cortical interneuron progenitors into the adult mouse hippocampus resulted in robust survival, migration, and further differentiation. At 30 days post-transplantation, grafted cells expressed the cortical interneuron markers FOXG1, GABA, and SST, extended long neuronal processes, and formed presynaptic puncta opposed to host neurons. These observations indicate that hiNSC-derived interneurons can integrate into host neural circuitry, supporting their potential for cell-based therapy. Due to the potential tumorigenicity of hiPSCs and hESCs, their derivative cell products must attain fully mature phenotypes. However, for cell therapy purposes, these developing migratory cortical interneurons may prove more beneficial than fully mature neurons, as these developing cells are more susceptible to host microenvironmental factors than fully mature neurons with complex neurites, and are more capable of migration, adjustment, and optimal integration into host circuits [55]. Compared with more immature progenitor cells, they are also better suited for cell therapy, as they no longer contain proliferative cells, ensuring product safety [9,55]. Given that hiNSCs have already been clinically validated for safety in Parkinson’s disease patients [31], our protocol offers a translational-ready platform for cortical interneuron production, which is not directly available from hiPSC/ESC-derived products due to residual pluripotency concerns.

Several limitations should be acknowledged. First, while we show marker expression and GABA positivity, electrophysiological recordings (e.g., patch-clamp) are needed to confirm mature fast-spiking interneuron properties, action potential firing patterns, and inhibitory synaptic currents. Second, although transplanted cells survive, migrate, and express interneuron markers at 4 weeks, behavioral rescue, seizure suppression, or circuit-level functional effects in disease models have not been demonstrated. These functional validations are essential for therapeutic translation and are ongoing in our laboratory. Third, the long-term safety and efficacy of hiNSC-derived interneurons in vivo require further assessment beyond the 4-week timepoint.

Overall, in our study, we adapted and optimized these strategies specifically for hiNSCs, modifying critical parameters such as the timing and duration of SMAD, WNT, and SHH pathway modulation. While the overall differentiation efficiency is comparable to previous hiPSC/ESC protocols, our approach substantially shortens the induction period, reduces culture costs, and produces cells at a developmental stage potentially advantageous for translational and clinical applications. This work will provide critical tools for effectively utilizing hiNSC-derived cortical interneuron progenitor cells for cell-based therapies.

## 4. Materials and Methods

### 4.1. hiNSCs and hiPSCs Culture and Differentiation

hiNSCs were generated from human peripheral blood mononuclear cells (PBMCs) by episomal vector reprogramming and subsequent neural induction [30,56]. Briefly, PBMCs were transduced with Sendai virus encoding *OCT3/4*, *SOX2*, *KLF4*, and *c-MYC*, then plated on PDL/laminin-coated dishes in hiNSC medium. Epithelium-like clones were manually picked between day 32–35, and residual virus was inactivated by culturing at 39 °C for one week at passage 3. hiNSCs were maintained in hiNSC medium and dissociated with Accutase (Innovative Cell Technologies, San Diego, CA, USA) for differentiation and passaging. hiNSC medium consisted of basic culture medium supplemented with 10 ng/mL human recombinant LIF (hrLIF, Millipore, Burlington, MA, USA), 3 µM CHIR99021 (Gene Operation, Ann Arbor, MI, USA), and 2 µM SB431542 (Gene Operation). As shown in Table 1, the basic culture medium comprised 48% DMEM/F12 medium (Gibco, Waltham, MA, USA), 48% Neurobasal medium (Gibco), 1% B27 supplement (Gibco), 1% N2 supplement (Thermo Fisher, Waltham, MA, USA), 1% non-essential amino acids (NEAAs, Gibco), and 1% GlutaMax (Gibco). hiPSCs were purchased from Cellapy (Beijing, China) and maintained in Essential 8 (E8) medium (Gibco) on matrigel-coated dishes.

For differentiation into cortical GABAergic progenitors (GABAPs), both cell types were subjected to the same protocol. Cells were dissociated into single cells using Accutase and seeded at a density of 10,000 cells per well in low-attachment 96-well plates with 150 μL hiNSC medium for 2 days. Cortical GABAergic progenitors differentiation was initiated using phase 1 differentiation medium consisting of basic culture medium supplemented with 200 μM ascorbic acid (Sigma-Aldrich, St. Louis, MO, USA) and 55 μM β-mercaptoethanol (Life Technologies, Carlsbad, CA, USA). Small-molecule compounds were added as follows: 10 µM SB431542 (Gene Operation), 250 nM LDN193189 (TargetMol, Boston, MA, USA), 10 µM XAV939 (TargetMol), and 100 nM SAG (TargetMol). For neural induction, cells were treated with phase 2 differentiation medium, consisting of basic culture medium supplemented with 2 µM PD0325901 (TargetMol). To compare different culture conditions, cells were either cultured statically in PDL/laminin-coated dishes or maintained in suspension on an orbital shaker at 80 rpm starting from phase 2. From the third stage onwards, cells were maintained in basic culture medium containing 2 µM PD0325901 (TargetMol), 2 µM PD0332991 (TargetMol), and 10 µM DAPT (TargetMol). For long-term culture, cells were cultured in basic culture medium supplemented with 20 ng/mL brain-derived neurotrophic factor (BDNF, Peprotech, Rocky Hill, NJ, USA), 10 ng/mL glial cell line-derived neurotrophic factor (GDNF, Peprotech), and 500 μM dibutyryl-cAMP (db-cAMP, Sigma-Aldrich). The medium was carefully replaced every two days. A detailed list of all small-molecule compounds, growth factors, and supplements used for differentiation, including their concentrations, suppliers, catalog numbers, and primary functions, is provided in Supplemental Table S1.

In all transplantation experiments, cells were washed thoroughly by repeated centrifugation to remove dead cells and debris, yielding cell preparations with >80% viability. Cell suspensions were prepared in long-term culture medium prior to grafting.

### 4.2. Cell Viability Assay

To assess the proliferative capacity of cortical interneurons, cells were dissociated using Accutase on days 7 and 10 of differentiation. Following 5 min incubation at 37 °C, both suspension-cultured spheres and adherent cells were mechanically triturated to generate single-cell suspensions. An equal volume of DMEM medium was added to neutralize the Accutase. A 10 μL aliquot of cell suspension was mixed with an equal volume of 0.4% trypan blue solution, and viable and non-viable cells were counted using a hemocytometer to determine viable cell concentration.

Overall metabolic activity was assessed using the Cell Counting Kit-8 (CCK-8) assay. A standard curve was first generated by plotting cell number on the x-axis against optical density (OD) values on the y-axis. This standard curve was used to optimize cell density and incubation time for subsequent CCK-8 experiments. Cells were seeded at densities of 1000, 5000, and 10,000 cells per well (100 μL per well in 96-well plates), with five technical replicates for each density. After 2–4 h incubation to allow cell attachment, 10 μL of CCK-8 reagent was added to each well, and OD values at 450 nm were measured at 0.5, 1, 2, and 4 h post-addition using a microplate reader. Following optimization, metabolic activity was quantified in cells at differentiation days 7 and 10 according to the manufacturer’s instructions. Undifferentiated hiNSCs maintained in expansion medium served as controls.

### 4.3. Liquid Chromatography–Tandem Mass Spectrometry (LC-MS/MS) Analysis

Cellular inhibitory neurotransmitter GABA content was quantified by LC-MS/MS in hiNSC-derived cells and undifferentiated hiNSCs (as controls) after 60 days of differentiation. For stimulated GABA release measurements, organoid culture medium was first replaced with HBSS (Gibco, Waltham, MA, USA) (3 mL per well) and incubated at 37 °C for 30 min. To prevent neurotransmitter degradation, the supernatant was subsequently replaced with 3 mL high-potassium HBSS medium (60 mM KCl and 82 mM NaCl) containing the GABA reuptake inhibitor tiagabine (5 μM, TargetMol, T2407) and the 4-aminobutyrate transaminase inhibitor vigabatrin (3 mM, TargetMol, T0128), and incubated for 45 min at 37 °C. Then, 180 μL of high-potassium medium from each well was transferred to amber Eppendorf tubes containing 20 μL of 1 N perchloric acid (Merck, Rahway, NJ, USA) on ice and stored at −80 °C until analysis.

For spontaneous GABA secretion measurements, 200 μL of culture supernatant was directly collected, transferred to amber Eppendorf tubes, and stored at −80 °C until multiple reaction monitoring (MRM) analysis.

Sample preparation and chromatographic conditions. Samples were mixed with a methanol/acetonitrile aqueous solution and centrifuged at 4 °C. The supernatant was transferred to autosampler vials for LC-MS/MS analysis. Chromatographic separation was performed on a Waters ACQUITY UPLC HSS T3 C18 column (Milford, MA, USA) (1.8 μm, 100 mm × 2.1 mm i.d.). The column temperature was maintained at 40 °C. Mobile phase A consisted of ultrapure water containing 0.1% formic acid, and mobile phase B consisted of acetonitrile containing 0.1% formic acid. The gradient elution program was as follows: 0 min, 95:5 (A/B, *v*/*v*); 8 min, 5:95 (A/B, *v*/*v*); 9.5 min, 5:95 (A/B, *v*/*v*); 9.6 min, 95:5 (A/B, *v*/*v*); 12 min, 95:5 (A/B, *v*/*v*). The flow rate was 0.35 mL/min, and the injection volume was 2 μL.

Mass spectrometry analysis. Mass spectrometric analysis was performed using an ultra-performance liquid chromatography system (ExionLC™ AD, SCIEX, Framingham, MA, USA, https://sciex.com.cn/) coupled with a tandem mass spectrometry system (QTRAP^®^ 6500+, SCIEX, https://sciex.com.cn/) operated in MRM mode.

Quantification and quality control. Quantification was performed using the internal standard method. Calibration curves were prepared using a series of analyte standards at known concentrations with a fixed concentration of internal standard. Linear regression of the peak area ratio (analyte/internal standard) versus concentration was performed to generate calibration curve equations. All calibration curves exhibited excellent linearity (R^2^ > 0.99). Each analytical batch included blank samples and quality control samples at different concentration levels to ensure accuracy and precision throughout the analytical process.

### 4.4. In Vitro Cell Migration Assay

For Matrigel-based and two-dimensional migration analysis, suspension-cultured spheres or adherent inhibitory interneuron precursors at differentiation on day 14 were embedded in Matrigel and seeded onto poly-D-lysine (PDL) and laminin-coated 6-well plates.

### 4.5. RNA Sequencing (RNA-Seq)

Library preparation and sequencing were performed alongside the isolation of total RNA using the E.Z.N.A. Total RNA Kit I (Omega Bio-tek, Norcross, GA, USA) was used, and directional cDNA libraries were constructed using the SMARTer Stranded Total RNA-Seq Kit V2 (Clontech/Takara, Mountain View, CA, USA) according to the manufacturer’s instructions. Libraries were sequenced on an Illumina HiSeq 3000 platform (San Diego, CA, USA) using single-end 50 bp reads (1 × 50 configuration).

Read alignment and quantification: RNA-seq reads were aligned to the human reference genome (hg38/GRCh38) using the STAR aligner version 2.5.4b with default parameters. Gene-level read counts were quantified from uniquely aligned unambiguous reads using Subread featureCounts version 1.4.6, with gene annotations based on GENCODE release V27.

### 4.6. Differential Expression Analysis and Data Visualization

All gene-level read counts were imported into the R/Bioconductor package DESeq2 (version 1.x). Genes with counts per million (CPM) > 1.0 in at least one sample were retained for downstream analysis. Count data were normalized and regularized log-transformed using the rlog function in DESeq2. Differential gene expression (DGE) analysis was performed using DESeq2 with default parameters. Adjusted *p*-values were calculated using the Benjamini–Hochberg method to control the false discovery rate (FDR). Genes with an absolute log_2_ fold change ≥ 1 (corresponding to a 2-fold change) and FDR < 0.01 were considered significantly differentially expressed.

Principal component analysis (PCA) was performed using the plotPCA function from the DESeq2 package, and results were visualized using ggplot2 (v3.4.4). Differentially expressed gene lists from all pairwise comparisons were merged and annotated with their corresponding transcripts per million (TPM) values for cross-condition comparisons. The functions enrichGO, KEGG, and GSEA from the R package clusterProfiler (v4.8.3) were employed to conduct enrichment analysis. We performed GSEA on the gene sets sorted by log2 fold change between the compared groups. A representative subset of the GO terms sorted by semantic similarity was visualized by the SRplot Web server (available at https://www.bioinformatics.com.cn/).

### 4.7. Cell Transplantation into the Right Hippocampus of Naive Mice

All animal experiments were conducted in accordance with the guidelines for the Care and Use of Laboratory Animals established by the Beijing Association for Laboratory Animal Science and approved by the Ethics Committee of Xuanwu Hospital, Capital Medical University (XW-20210520-1). Mice were housed under a 12 h light/dark cycle with water and food available ad libitum. Male mice were selected for all experiments, as estrogen affects epilepsy model stability and seizure consistency.

NPG (NOD.Cg-*Prkdc^scid^ Il2rg^tm1^*/Vst) male mice, aged 6–8 weeks, were obtained from VitalStar Biotechnology (Beijing, China) (with 5 mice per experimental group). Transplantation of hiNSC-derived inhibitory interneurons or vehicle control (serum-free basal medium) was performed in naive mice. Cells (1 × 10^6^ cells per mouse) were injected at three different depths using stereotaxic coordinates (AP −3.30 mm, ML +3.3 mm, DV −3.2 mm, −3.7 mm, and −4.2 mm from bregma) with a 26-gauge Hamilton microsyringe (10 μL) connected to a microinjection system (Reward Life Technology, Shenzhen, China). The injection rate was 0.5 μL/min, and the needle was left in place for an additional 5 min before slow retraction. Control mice received equivalent volumes of vehicle solution at the same stereotaxic coordinates.

Mice (average body weight 23–24 g) were anesthetized with isoflurane (induction 3%; maintenance 1.5–2%) and placed in a stereotaxic frame on a heating pad to maintain body temperature at 37 °C. Total anesthesia duration was kept below 1 h to minimize the risk of post-anesthetic seizures. Animals were continuously monitored during recovery. Mice received 400 μL of lactated Ringer’s solution subcutaneously for fluid resuscitation during and after surgery. Animals showing signs of distress or failure to thrive were humanely euthanized. Mortality during and within the first few days following post-surgery was less than 10%.

### 4.8. Immunofluorescence Assay

For immunofluorescence analysis of cultured cells, cells were plated on poly-D-lysine (PDL; 10 μg/mL) and laminin (10 μg/mL)-coated coverslips. Cells were fixed with 4% paraformaldehyde (PFA) in phosphate-buffered saline (PBS) for 10 min at room temperature, followed by two washes with PBS (5 min each). Fixed cells were permeabilized and blocked with blocking buffer (3% donkey serum and 0.3% Triton X-100 in PBS) for 1 h at room temperature. Cells were then incubated with primary antibodies diluted in antibody dilution buffer (1% donkey serum and 0.3% Triton X-100 in PBS) overnight at 4 °C. The use of the primary antibodies is summarized in Table 2. After three washes with PBS, cells were incubated with fluorophore-conjugated secondary antibodies diluted in antibody dilution buffer for 1 h at room temperature in the dark. Nuclei were counterstained with 4′,6-diamidino-2-phenylindole (DAPI; 1 μg/mL) for 15 min at room temperature. Coverslips were mounted on glass slides using fluorescence mounting medium and imaged using confocal microscopy (Leica STELLARIS 5, Wetzlar, Hesse, Germany).

### 4.9. Tissue Processing and Immunofluorescence Staining

Mice were deeply anesthetized and transcardially perfused with 50 mL of 0.9% saline followed by 100 mL of ice-cold 4% PFA in PBS (pH 7.4). Brains were rapidly dissected and post-fixed in 4% PFA overnight at 4 °C, then cryoprotected by sequential immersion in 20% sucrose (in 0.1 M phosphate buffer) overnight and 30% sucrose until tissues sank. Brains were embedded in optimal cutting temperature (OCT) compound, and coronal sections (20 μm thickness) were cut on a cryostat and collected as 12 serial sets.

For immunohistochemical staining, free-floating sections were blocked with 3% donkey serum in 0.3% Triton X-100/PBS for 2 h at room temperature, and then incubated with primary antibodies (Table 1) overnight at 4 °C. After washing, sections were incubated with appropriate fluorophore-conjugated secondary antibodies for 2 h at room temperature, counterstained with DAPI, mounted on glass slides, and coverslipped with fluorescence mounting medium. Images were acquired using confocal microscopy (Leica STELLARIS 5).

### 4.10. Statistical Analysis

All data are presented as mean ± standard error of the mean (SEM) and were analyzed using GraphPad Prism version 9.0 (GraphPad Software, San Diego, CA, USA). For comparisons between two groups, parametric data were analyzed using an unpaired two-tailed Student’s *t*-test, while non-parametric data were analyzed using the Mann–Whitney *U* test. For comparisons among three or more groups, parametric data were analyzed by one-way analysis of variance (ANOVA) followed by Bonferroni’s post hoc test for multiple comparisons, and non-parametric data were analyzed using the Kruskal–Wallis test followed by Dunn’s post hoc test. Statistical significance was set at *p* < 0.05. Sample sizes, specific statistical tests, and exact *p* values are reported in the figure legends.

## Figures and Tables

**Table 1:** Composition of basic culture medium.

Component	Final Concentration	Manufacturer
DMEM/F12 medium	48% (*v*/*v*)	Gibco
Neurobasal medium	48% (*v*/*v*)	Gibco
B27 supplement	1% (*v*/*v*)	Gibco
N2 supplement	1% (*v*/*v*)	Thermo Fisher
Non-essential amino acids (NEAA)	1% (*v*/*v*)	Gibco
GlutaMax	1% (*v*/*v*)	Gibco

**Table 2:** Antibody list used in the experiments.

Antibody	Species	Dilution	Source
Tuj1	Mouse	1/500	Santa Cruz (Dallas, TX, USA)
GABA Rabbit	Rabbit	1/500	Sigma (St. Louis, MO, USA)
LHX6	Mouse	1/500	Santa Cruz (Dallas, TX, USA)
Nkx2.1	Mouse	1/500	Proteintech (Rosemont, IL, USA)
FOXG1	Rabbit	1/500	Abcam (Cambridge, Cambridgeshire, UK)
STEM121	Mouse	1/500	Takara (Kusatsu, Shiga, Japan)
Parvalbumin	Rabbit	1/500	Abcam (Cambridge, Cambridgeshire, UK)
SST	Rabbit	1/500	Abcam (Cambridge, Cambridgeshire, UK)
Ki67	Mouse	1/500	Millipore (Burlington, MA, USA)
GFAP	Mouse	1/500	Santa Cruz (Dallas, TX, USA)
VGLUT	Rabbit	1/500	Abclonal (Wuhan, Hubei, China)
VGAT	Rabbit	1/500	Synaptic Systems (Göttingen, Lower Saxony, Germany)
GABA-A receptor alpha1	Mouse	1/500	Synaptic Systems (Göttingen, Lower Saxony, Germany)
FOXA2	Mouse	1/500	Abcam (Cambridge, Cambridgeshire, UK)
PAX6	Mouse	1/500	DSHB (Iowa City, IA, USA)
GAD1	Rabbit	1/500	Sigma (St. Louis, MO, USA)

## Data Availability

All data are present in the manuscript. The data that support the findings of this study are available from the corresponding author upon reasonable request.

## Supplementary Materials

The following supporting information can be downloaded at: https://www.mdpi.com/article/10.3390/ijms27125194/s1.
