# Supplementary material for: Rapid and Robust Generation of Human Cortical Interneurons from Induced Neural Stem Cells
Source: Int J Mol Sci. 2026 Jun 8;27(12):5194. doi: 10.3390/ijms27125194 (PMC13299594; doi:10.3390/ijms27125194)
Supplement: Supplementary file 1 [file ijms-27-05194-s001.zip › ijms-4288527-supplementary.pdf]

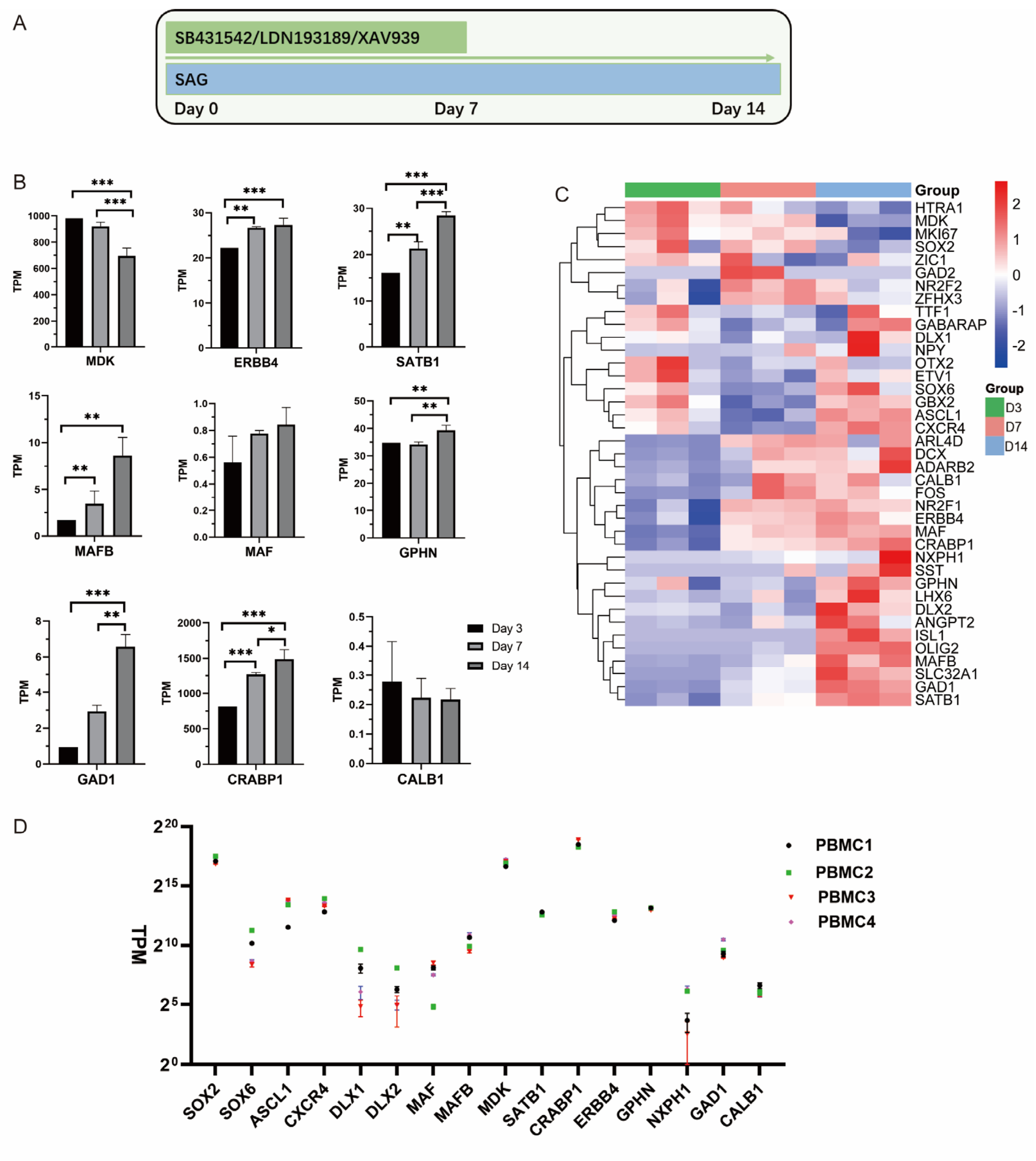

Supplemental Figure S1. Transcriptomic profiling of hiNSC differentiation under short-term SMAD and WNT inhibition.

(**A**-**B**) Differentiation workflow (**A**) and transcriptomic analysis (**B**) at day 14 with 7-day SMAD and WNT inhibition. (**C**) Heatmap of stage-specific gene expression across key time points during hiNSC differentiation. Colors represent normalized expression values for selected markers associated with interneuron lineage specification and neural progenitor identity. (**D**) Relative expression of stage-specific genes in four donor-specific hiNSC lines at day 14 post differentiation. PBMC1–4 denote hiNSC lines derived from four independent PBMC donors. Expression levels were normalized to housekeeping genes.

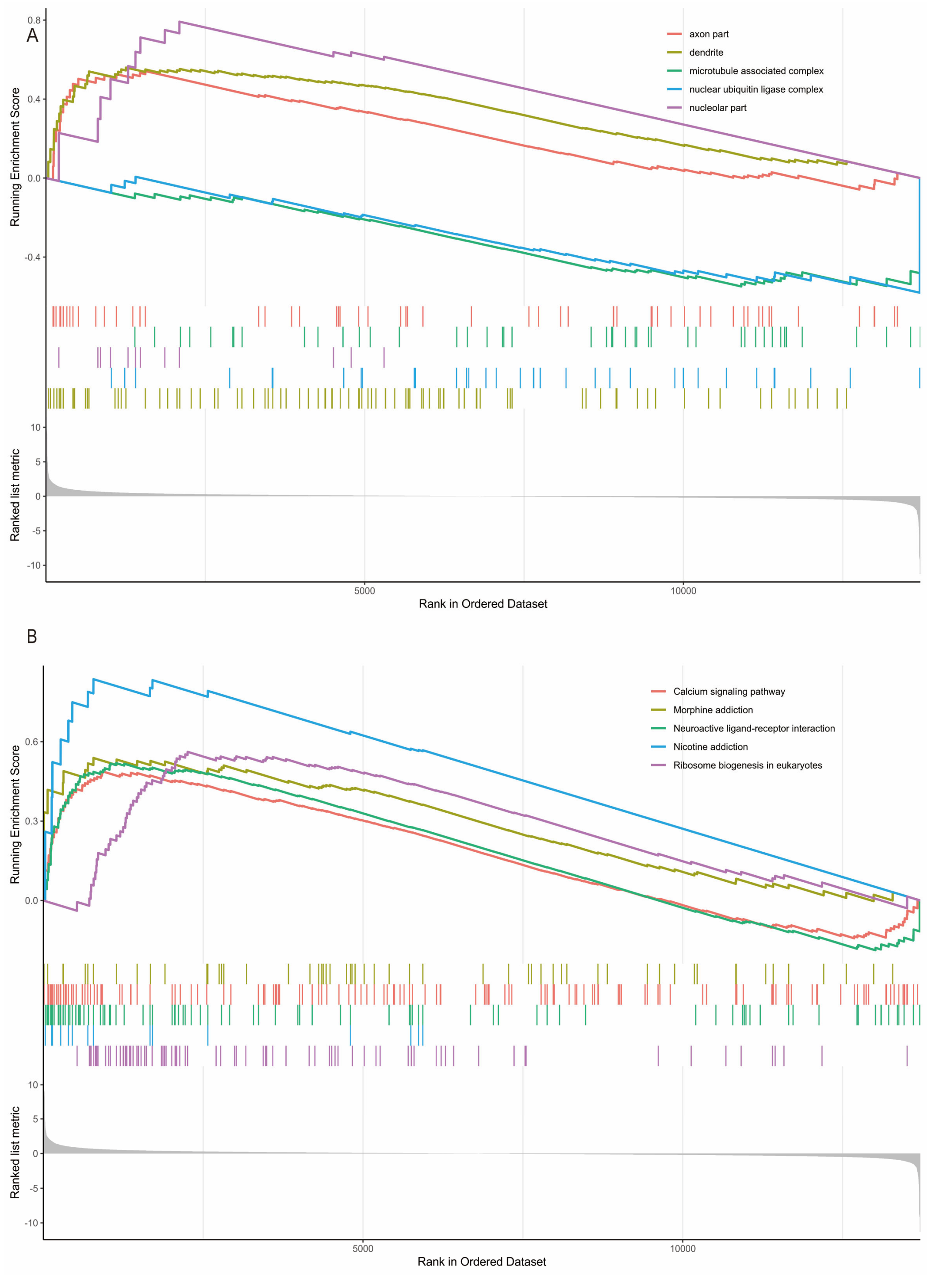

Supplemental Figure S2. GSEA of RNA-seq data comparing day 14 versus day 7 SAG-treated hiNSCs.

(**A**) Enrichment plots for the top five Cellular Component terms: axon part, dendrite, microtubule-associated complex, nuclear ubiquitin ligase complex, nucleolar part.

(**B**) Enrichment plots for the top five KEGG pathways: Calcium signaling pathway, Morphine

addiction, Neuroactive ligand-receptor interaction, Nicotine addiction, Ribosome biogenesis in eukaryotes.

Normalized enrichment score (NES), nominal P-value, and FDR are indicated within each plot. Gene sets with FDR < 0.25 were considered significantly enriched. Positive NES indicates enrichment in 14-day SAG-treated hiNSCs; negative NES indicates enrichment in 7-day SAG-treated hiNSCs.

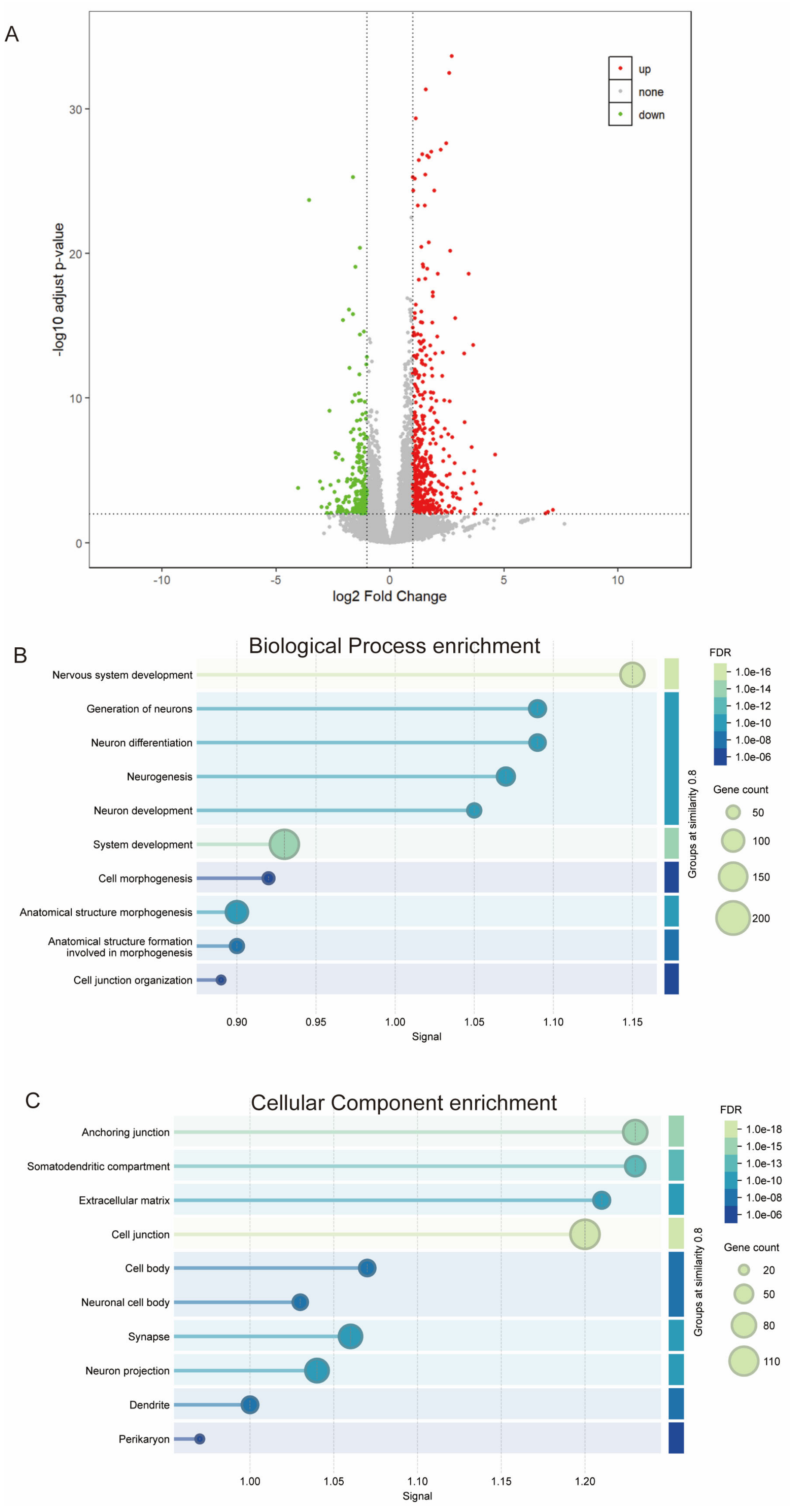
A
up
none
down
-log10 adjust p-value
log2 Fold Change
B
Biological Process enrichment
Nervous system development
Generation of neurons
Neuron differentiation
Neurogenesis
Neuron development
System development
Cell morphogenesis
Anatomical structure morphogenesis
Anatomical structure formation involved in morphogenesis
Cell junction organization
Signal
Groups at similarity 0.8
FDR
1.0e-16
1.0e-14
1.0e-12
1.0e-10
1.0e-08
1.0e-06
Gene count
50
100
150
200
C
Cellular Component enrichment
Anchoring junction
Somatodendritic compartment
Extracellular matrix
Cell junction
Cell body
Neuronal cell body
Synapse
Neuron projection
Dendrite
Perikaryon
Signal
Groups at similarity 0.8
FDR
1.0e-18
1.0e-15
1.0e-13
1.0e-10
1.0e-08
1.0e-06
Gene count
20
50
80
110

Supplemental Figure S3. Transcriptomic comparison of adherent vs. shaker cultures during hiNSC differentiation.

(**A**) Volcano plot showing differentially expressed genes (DEGs) upregulated at day 10 of induction in adherent versus shaker cultures. (**B**-**C**) GO enrichment analysis of upregulated DEGs at day 10 of induction in adherent cultures. Compared to shaker culture, adherent culture displayed a more advanced differentiation stage. (**B**) Biological Process and (**C**) Cellular Component categories are shown.

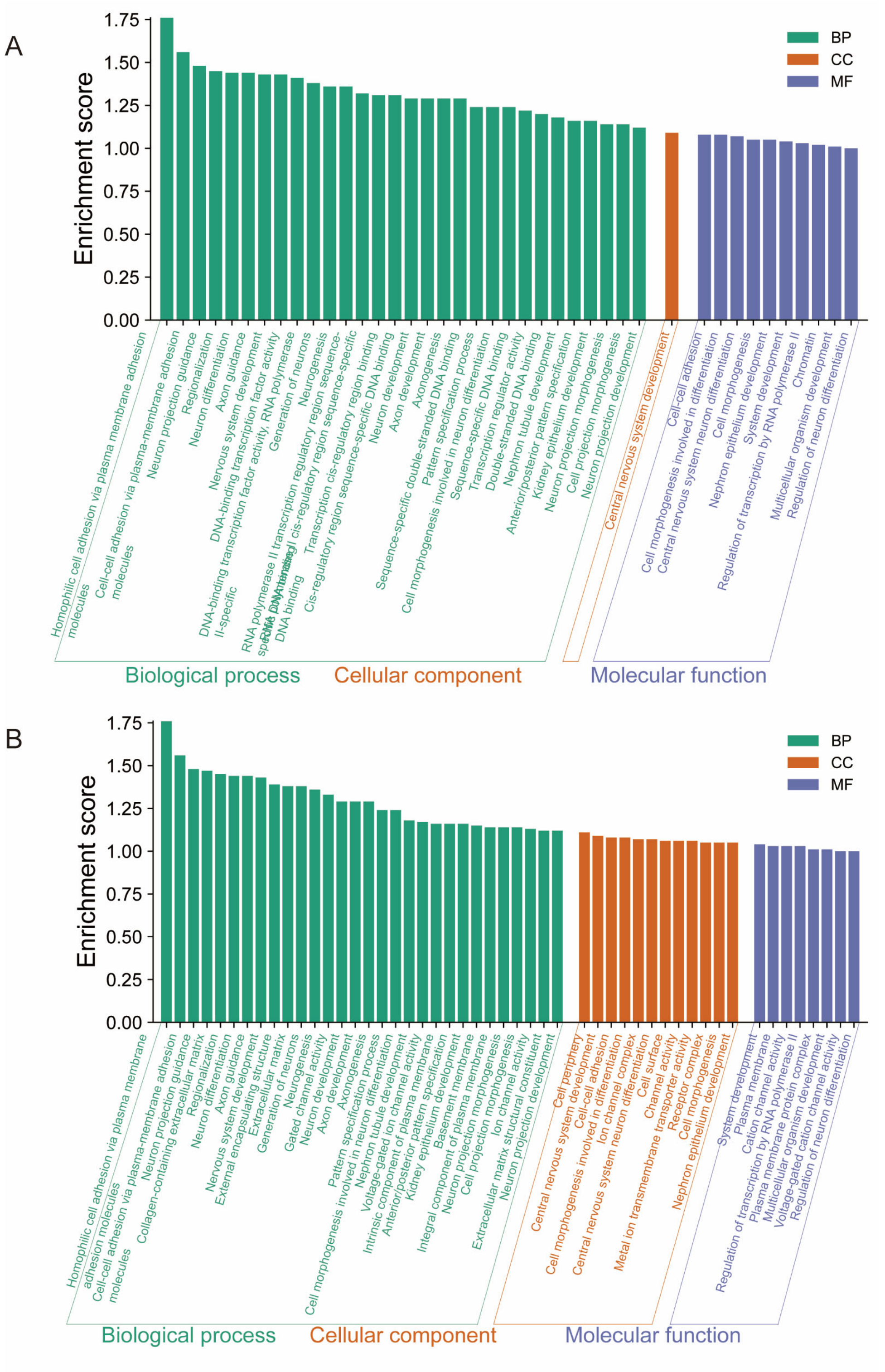

Supplemental Figure S4. Gene Ontology (GO) enrichment analysis of upregulated genes in hiNSC-derived cells (**A**) and hiPSC-derived cells (**B**) at day 14 of induction.

Supplemental Table S1. Cell differentiation-related molecules by culture stage.

Culture stage	Molecule/reagent	Final concentration	Manufacturer, catalog	Molecular function
hiNSC maintenance	hrLIF	10 ng/mL	Millipore, LIF1010	Maintains stem cell pluripotency
	CHIR99021	3 µM	Gene Operation, 04-0004	Wnt/β-catenin pathway activator
	SB431542	2 µM	Gene Operation, 04-0010-10mg	TGF-β/Activin/Nodal pathway inhibitor
Phase 1 (GABAP induction)	LDN193189	250 nM	TargetMol, T1852	BMP pathway inhibitor
	XAV939	10 µM	TargetMol, T2304	Wnt pathway inhibitor
	SAG	100 nM	TargetMol, T1587	Hedgehog pathway agonist
	SB431542	10 µM	Gene Operation	
	Ascorbic acid	200 µM	Sigma-Aldrich, A4544	Antioxidant, promotes neural differentiation
	β-mercaptoethanol	55 µM	Life Technologies, 21985023	Reducing agent, reduces oxidative stress
Phase 2 (neural induction)	SAG	100 nM	TargetMol	
	PD0325901	2 µM	TargetMol, T1327	MEK/ERK pathway inhibitor
Third stage culture	PD0325901	2 µM	TargetMol	
	PD0332991	2 µM	TargetMol, S1116	CDK4/6 inhibitor
	DAPT	10 µM	TargetMol, T1246	Notch pathway inhibitor
Long-term culture	BDNF	20 ng/mL	Peprotech, 450-02	Promotes neuronal survival and maturation
	GDNF	10 ng/mL	Peprotech, 450-	Neurotrophic

			10	factor
	db-cAMP	500 μM	Sigma-Aldrich, D0627	cAMP analog
